# Buffalo College of Physicians and Surgeons

**Published:** 1881-09

**Authors:** 


### Editorial.

### Buffalo College of Physicians and Surgeons.

As most of our readers are probably aware, there has been in operation within this city for some time past, an institution calling itself the "Buffalo College of Physicians and Surgeons." We have from time to time observed, in our various exchanges, notices of this college, which have been anything but complimentary to it, or to the fair city of Buffalo, within whose confines it is situated. Some have gone so far as to compare it to the notorious Buchanan College of Philadelphia. Certain developments in connection with the granting of diplomas to those manifestly unqualified, and which the College has since been constrained to revoke, add to the unenviable reputation of the institution. We have, therefore, deemed it proper to give the present facts in the case as we understand them.

In March 1879 a number of gentlemen formed a corporation under the title of the Buffalo College of Rational Medicine, basing their authority upon the well-known law of 1848, for the incorporation of benevolent, charitable and missionary societies. In less than three months thereafter a new charter was obtained and the name changed to the "Buffalo Homœopathic College of Physicians and Surgeons." Still again, about a year later, the name was once more changed and the word "Homœopathic" stricken off, leaving simply the title as it now stands, "The College of Physicians and Surgeons." The last change did not, it seems, require a new charter, but was made in accordance with an order obtained from the Supreme Court.

Under its latest name the College last winter gave one course of lectures, and in the spring furnished diplomas to a number of its graduates.

The Board of Censors of the Medical Society of the County of Erie having been instructed by said society to prosecute all persons practicing in violation of the new law, decided this to be a "useless task" so long as the college in question should be allowed unlimited and unrestricted power to grant diplomas. The attention of the Attorney-General was therefore called to the matter, and an order was issued by him directing the determination of the question at issue—the legality of the charter founded upon the law of 1848 and its amendments. The question was argued and submitted and the decision of the Hon. George Barker, Judge of the Supreme Court, is given as the leading article of the Journal.

